# “A passion to change the landscape and drive a renaissance”: The mRNA Hub at Afrigen as decolonial aspiration

**DOI:** 10.3389/fpubh.2022.1065993

**Published:** 2022-11-28

**Authors:** Lauren Paremoer, Anne Pollock

**Affiliations:** ^1^Department of Political Studies, University of Cape Town, Cape Town, South Africa; ^2^Department of Global Health and Social Medicine, King's College London, London, United Kingdom

**Keywords:** vaccine inequality, mRNA Hub, decolonial science, intellectual property, dignity, solidarity, interdependence

## Abstract

In global conversations about COVID-19 vaccine access, Africa has overwhelmingly been characterized as a site of lack. However, the successful reverse engineering of the Moderna vaccine by Afrigen at the mRNA Hub in Cape Town marks a more hopeful path forward. This paper introduces the mRNA Hub and puts it into context of broader decolonial aspirations for African futures in the face of rich countries' disregard. Highlighting ways in which Afrigen's Managing Director's articulations of the endeavor resonate with historical and contemporary calls to dismantle the unequal global order, we argue that the mRNA Hub provides an example of the potential for decolonial solidarity in the post/pandemic period.

## Introduction

In May 2020, the Trump administration launched Operation Warp Speed (OWS) (1). Its name, borrowing from science fiction, is reminiscent of other government programmes aimed at realizing barely possible technologies of salvation and protection (Ronald Reagan's Star Wars programme comes to mind). OWS invested billions of dollars of US public funding into accelerating the development of COVID-19 vaccines, therapeutics, and diagnostics. One year later, the World Health Organization (WHO) announced a program aimed at building the capacity of low- and middle-income states (LMICs) to produce mRNA based COVID-19 vaccines. The program, as ambitious as OWS, was less spectacularly named. Using the language of terrestrial, analog technologies, the WHO indicated this research and development (R&D) initiative would be organized around a Hub-and-Spoke model (2).

Both interventions have been incredibly successful. OWS was crucial in ensuring that safe and effective COVID-19 vaccines were brought to market within months rather than years (3). In June 2021 a South African consortium including the BioVac Institute and Afrigen Biologics and Vaccines (“Afrigen”) were announced as key participants in a team that would build the WHO mRNA Hub (4). Although the original WHO vision was for the originator companies to assist in tech transfer and capacity building, the lack of cooperation from Moderna or Pfizer led to the alternative approach of reverse engineering (5). By February 2022 Afrigen announced that it had replicated the Moderna COVID-19 vaccine, using only publicly available information, with no cooperation from Moderna, and under threat of potential intellectual property rights (IPR) litigation should Moderna feel the Hub's activities violated the three patents on its technologies that Moderna had been granted by the South African government (6). Technical bodies including WHO (7), Medicines Patent Pool (MPP) (8), and the Vaccine Research Center (VRC) at the USA's National Institute of Allergy and Infectious Diseases' (NIAID's) (9) have affirmed that the Hub has the required local capacity, the know-how, and expertise to replicate and produce mRNA technologies and support tech transfer to the spokes.

If narratively the Hub-and-Spoke model might articulate a rather mundane counterpoint to the sensational Operation Warp Speed, we argue that it does important decolonial work both imaginatively and materially. The mRNA Hub instantiates for post-coronavirus Africa what Ghanaian literary scholar Rogers Asempasah has characterized as vital for postcolonial liberation, by “transcending the fetishization or allure of Europe and America as the horizons of hope or possibility” (10). Asempasah is writing about literary imagination, but his insight is in dialogue with and relevant to scientific imagination as well: “what Africa requires in this era of waiting for vaccines from the West, or turning to others for the resolution of Africa's problems, is the decolonization of the ‘beyond”' (10).

## Confronting big pharma's disregard for Africa

Despite its spectacular success, some have considered the ultimate goal of the Hub – ensuring that LMICs have their own mRNA production facilities – to be a ludicrous notion. While there might be legitimate reasons to argue for the pursuit of alternatives to mRNA-based technologies in African contexts, for example because plant-based manufacturing facilities are cheaper to set up and protein-based vaccines avoid the need for ultra-cold storage (11), the most prominent objections to the Hub have instead been patronizing and dismissive. Comparing patented COVID-19 vaccines to high-end “lifestyle products,” and implicitly accusing the Hub of “pirating” Moderna's vaccine, Moderna Chief Executive Officer Stéphane Bancel has argued.

“We've never helped them [Afrigen] develop it or make it. We have never certified their quality control. They are claiming it's a copy of Moderna's product. I don't know”; “It is like when somebody makes a copy of a Louis Vuitton bag. Does it look like a Louis Vuitton bag? Does it last like a Louis Vuitton bag? I don't know” (12).

He has argued that collaboration with the Hub is “not a good use of our [Moderna's] time” because,

“If I need to send engineers to the mRNA hub, I need to be explaining which product I'm not going to do or delay…Do you want me to delay a cancer drug or a rare disease drug for kids or a CMV [cytomegalovirus] vaccine to prevent birth defects in a pregnant woman getting infected by this virus?” (13).

In addition, Bancel and Pfizer CEO Albert Bourla have both argued that their production capacity is sufficient for supplying global demand for COVID-19 vaccines, and as such a new producer like the Hub is, in Bancel's words, “nice to have, not a must have” (13). Opposition to the Hub has not only been rhetorical, or aimed at shaping public opinion. These powerful pharma actors have also sought to intervene on policy. In February 2022 the *BMJ* reported that BioNTech, who along with Pfizer has patented the Comirnaty vaccine, hired a consultancy, the kENUP Foundation. In a document submitted to South African government officials, kENUP argued that the Hub was unlikely to be successful, would probably infringe on patents, and told officials that “The WHO Vaccine Technology Transfer Hub's project of copying the manufacturing process of Moderna's COVID-19 vaccine should be terminated immediately” (14).

For pharmaceutical industry leaders, negativity toward the Hub is continuous with the broader resource-hoarding that they have pursued through patents, as they have categorically refused to contribute to patent pools or consider any Trade Related Aspects of Intellectual Property Rights (TRIPS) waivers even in the COVID-19 context of massive public investment on the one hand and global crisis on the other. When Pfizer chief Albert Bourla derided even voluntary patent sharing as “nonsense” and “dangerous,” (15) his sentiments suggest that pharmaceutical sovereignty in and solidarity with LMICs' scientific endeavors are equally impossible dreams. One could argue that, in opposing the TRIPS waiver request, the governments of the EU, Switzerland, and UK, deepened this idea that it is “impossible” for those outside the pharmaceutical metropoles to fundamentally change the terms of scientific and commercial engagement in pharmaceutical R&D.

Yet, despite the failure of the waiver request at the Twelfth World Trade Association Ministerial Conference (MC12) in June 2022, the mRNA Hub and the TRIPS waiver campaign helped re-ignite ideas last prioritized in the decolonizing period of the 1950's and 1960's. The financialization and globalization of the pharmaceutical sector in recent decades had seemingly eviscerated high-level political commitments to securing infrastructures for local production of essential medicines, development of new medical technologies that match the disease profiles and infrastructure constraints of LMICs, and investment of LMIC public funding in new industries. However, with the launch of the mRNA Hub initiative, the notion of public stewardship to ensure pharmaceutical sovereignty in LMICs was not only endorsed in principle, but was actively being pursued.

## African scientific capacity and/as African dignity

Afrigen challenges the common-sense notion that local production of essential medicines, especially using new platforms like mRNA, is an impossibility. It plans to use the mRNA platform it has developed to create vaccines for diseases that absorb much of South Africa's public health budget (TB and HIV/AIDS) and vaccines for neglected diseases affecting the African continent (e.g., Ebola, Zika, Lassa Fever, and Malaria). It also seeks to create new technologies aimed at creating “in-house innovation[s] by South African scientists, engineers and other stakeholders” in order to “develop second generation mRNA vaccines, suitable for the local markets and environments of LMICs” (16). This would include, “using different lipids and processing with different ratios, thereby developing [their] own knowledge base to provide the freedom to operate” (16). This would translate into new, locally-owned IPR to mRNA technologies that Afrigen hopes will allow the Hub to avoid patent litigation.

Before Afrigen's success, these kinds of aspirations would have read like nostalgia for a long-lost Third Worldist future where technical assistance is explicitly tied to a broader political project of repair; specifically, repairing the “denial of dignity” (17) brought about through colonialism and imperialism, and their afterlives. Remarkably these are the terms in which Petro Terblanche, Afrigen's Managing Director, understands the significance of the Hub's efforts. She describes the mRNA Hub and Spoke model as something more than a duplication effort. In her view it is a creative process involving scientific virtuosos from all over Africa, Latin America and Asia that have an existential reference point – avoiding human suffering – as their lodestar,

“If you were here and you were part of the void, you would have a different view [to Bancel]. If you have a passion for low- and middle-income countries, if you really have a passion to change the landscape and drive a renaissance, you will have a different view”; “For Stéphane Bancel, sitting in Boston, I can't believe that he could have a passion for something like this. And that's why this, for him, it's just a nuisance. But we are in it. We are Africans. For us, it is a passion” (18).

Terblanche has been emphatic about the originality of the Hub's scientific work. She has argued that it is not engaged in reverse engineering, “which implies that we've taken the actual vaccine, analyzed that and worked backwards to meet the qualities and the composition of what we have analyzed” (5). Instead, she argues that the Hub uses a process of “forward integration” that is based on “innovating from a sequence” (5). Using the sequence for Moderna's Vaccine 1273, which is in the public domain, Terblanche points out that the Hub proceeded to make “the plasmid using our own scientific knowledge base, our own instruments, and our own process mapping; we made the pDNA, we linearized and purified RNA and we encapsulated it” (5). In a context of global health partnerships in which “lopsidedness and ephemerality” is the norm (19), the agenda that Terblanche articulates is notable for its commitment to Africa and to the future.

## The solidarity-sovereignty nexus

On this view, science transforms nightmares into dreamscapes. The decolonizing aspirations of the mRNA Hub might be seen as continuous with forms of solidarity that were prominent in the 1970's but have since become marginalized. The connections drawn between the political marginalization of LMICs, technological progress (“renaissance” in Terblanche's words) and scientific solidarity evoke the language of the Declaration on the Establishment of a New International Economic Order (NIEO), which was adopted at the Sixth Special Session of the UN General Assembly on May 1, 1974. It frames technological progress as something that should improve the welfare of “the community of free peoples,” and calls for the dismantling of “the remaining vestiges of alien and colonial domination, foreign occupation, racial discrimination, apartheid and neo-colonialism in all its forms” (20). On this basis then the NIEO demands the “active, full and equal participation of the developing countries in the formulation and application of all decisions that concern the international community” (20). More specifically, with respect to technology transfer, it calls for the creation of “an international code of conduct for the transfer of technology corresponding to needs and conditions prevalent in developing countries” and “To expand significantly the assistance from developed to developing countries in research and development programmes and in the creation of suitable indigenous technology” [(20), Sections IV(a) and IV(c)].

Like the NIEO, 1978's Alma Ata Declaration on Primary Health Care explicitly argues that the right to health depends on the “full participation” of individuals, families and communities in decisions about the health technologies that should be made “universally acceptable” in their country, “at a cost that the community and country can afford to maintain at every stage of their development in the spirit of self-reliance and self-determination” (21). Like the NIEO Declaration, it embraces solidarity as distinct from charity. Both texts share a similar objective: liberating formerly colonized peoples from the continued relations of political, economic and scientific dependence they had been forced to endure after gaining formal independence (22). These sentiments are echoed in the words of South African President Cyril Ramaphosa, “It is not acceptable that Africa is consistently at the back of the queue in relation to access to medicines. While we appreciate the donations, they are never a sustainable mechanism to build resilience” (23).

It is worth underscoring that Ramaphosa's righteous plea is made not only on behalf of his country, but also on behalf of his continent. This is in stark contrast with the vaccine nationalism expressed by leaders of the world's richest countries, and is evocative of *third worldism* described by Andrew Nash as significantly flawed but nonetheless “as viable a model as we have of revolutionary internationalism as a living force, an ethos of consciously shaping ideas and actions not through pronouncements from above, but through building links of solidarity, exchanging ideas, developing common resources and engaging in collective action” (24). This ethos is something that we find inspiring in the example of the mRNA Hub. If the leaders of South Africa and other LMICs responded to the vaccine nationalism of rich countries merely with nationalisms of their own, this would not offer an authentically alternative articulation. By instead embracing a form of solidarity that is larger than the nation-state, Ramaphosa and others who support the Hub are recognizing the need for a form of sovereignty that recognizes and embraces interdependence, even as it seeks to restructure that interdependence on less unequal terms.

This sense of interdependence as an essential and valuable feature of both sovereignty and science challenges and destabilizes more dualistic conceptions of sovereignty and knowledge production as grounded in a state of total autonomy, all-knowingness, or, in the words of Francis Nyamnjoh, “completeness.” Instead, it grounds scientific knowledge production and the exercise of state sovereignty in a paradigm of “bridg[ing] various divides in the interest of the imperatives of living interconnections, nuances and complexities made possible or exacerbated by the evidence of mobilities and encounters” [(25), p. 7] – including the troubling mobilities and encounters catalyzed by the COVID-19 virus and the hoarding of technologies aimed at ameliorating its impact. As a result, this mode of sovereignty and science emphasizes “interconnections, interrelationships, interdependence, collaboration, coproduction and compassion …. Sameness, commonalities and possibilities ad infinitum, mean that everyone can act and be acted upon, just as anything can be subject and object of action, making power and weakness nimble-footed, fluid and situational, and giving life more a character of flux and interdependence than permanence. If hierarchies of social actors and actions exist, it is reassuring to know that nothing is permanent or singular about the nature, order and form of such hierarchies” [(25), pp. 8-9].

In light of this, one of the things that has been notable with the Hub is that it is South African but with pan-African solidarities that are material as well as rhetorical. Whereas previous South African scientific efforts have articulated the promise of scientists in South Africa taking leadership in finding “African solutions for African problems,” this has often been relatively disconnected from engagement with partners across the continent (26). In contrast, pan-African collaboration and sharing has been structured into the mRNA Hub's work from its earliest stages (27).

## Conclusion

Read from a decolonial perspective, the Hub opens up a profound challenge to the unjust colonial order decried so powerfully by Frantz Fanon, going beyond empty “piles of speeches on the equality of human beings” [(28), p. 89] to realize the “break up of the colonial world” [(28), p. 41]. It reframes Africa not as a place of lack (lacking expertise, infrastructure, resources, well-being, and good governance), but as home to African scientists and governments grounded in a scientific ethos driven by the need to work from existing constraints rather than ignoring them.

Harry Garuba and Benge Okot, writing on the practice of publishing literary texts that appear in more than one language, point out that the conceptual distinction between an “original” and a “translation” encourages us to think in vertical and hierarchical terms. They argue for a different conceptual approach: working within a conceptual field that thinks about difference in terms of lateral or horizontal relationships (29). This, they argue, allows works in different vernaculars to be located in relation to the particular but related “circuits of value” in which they accrue their meanings. Or in their own words, “Working with a conception of lateral textuality also allows us to uncouple the texts and to examine how each one is inserted into its particular literary tradition and the manner in which it partakes in its specific circuit of value” (29).

This conceptual orientation is reminiscent of Terblanche's assertion that Afrigen is not preoccupied with copying or translating the “original” (i.e., the Moderna vaccine) into a local variant. Her notion of “forward integration” suggests that the mRNA vaccine developed by the Hub is a distinct but related substance to Moderna's vaccine, and perhaps more importantly, that by virtue of its originality it is located outside the hegemonic “circuit of value” denoted by the TRIPS regime. Intellectual property is a circuit of value organized around the distinction between original/patented drugs vs. generic/copy/fake/counterfeit drugs (30).

In contrast to the Moderna mRNA vaccine, the work of the mRNA Hub at Afrigen circulates within a different circuit of value, where the notion of pharmaceutical research and development is tied to acknowledging interdependence and mutual aid/cooperation as a source of innovation and self-reliance, rather than a threat to dominance or sovereignty as autarky. When lack of access to COVID-19 vaccines in LMICs is framed as the failure of the richest countries to meet the needs of the other, it recapitulates a colonialist imaginary in a way that assumes the inevitability of international inequality (26). Thinking with the mRNA Hub offers a route to thinking the beyond.

## Data availability statement

The original contributions presented in the study are included in the article/supplementary material, further inquiries can be directed to the corresponding author/s.

## Author contributions

All authors listed have made a substantial, direct, and intellectual contribution to the work and approved it for publication.

## Funding

Support for the publishing costs has been provided by the COVID-19 Clinical Research Coalition, hosted by DNDi.

## Conflict of interest

The authors declare that the research was conducted in the absence of any commercial or financial relationships that could be construed as a potential conflict of interest.

## Publisher's note

All claims expressed in this article are solely those of the authors and do not necessarily represent those of their affiliated organizations, or those of the publisher, the editors and the reviewers. Any product that may be evaluated in this article, or claim that may be made by its manufacturer, is not guaranteed or endorsed by the publisher.
